# Using zebrafish to shed light on melanoma: an interview with Liz Patton

**DOI:** 10.1242/dmm.014340

**Published:** 2013-11

## Abstract

Liz Patton is a Senior Lecturer at the MRC Institute of Genetics and Molecular Medicine, University of Edinburgh, where she leads a research group whose goal is to understand melanocyte and melanoma development. Early on in her research career, Liz investigated cell cycle regulation in yeast and the implications for cancer, but now primarily exploits the zebrafish model to identify new pathways and therapeutic compounds relevant to melanoma. In this interview, Liz recalls some of her most exciting breakthroughs to date, discusses the advantages of zebrafish as a disease model and provides her perspectives on the current challenges in cancer research.

Elizabeth (Liz) Patton grew up in Halifax, Nova Scotia, Canada. She completed her undergraduate studies in microbiology and biology at King’s College and Dalhousie University in Halifax, during which she examined genetic pathways in the yeast cell cycle under the supervision of Gerry Johnston and Rick Singer. Inspired by her productive and enjoyable experiences in the lab, Liz embarked on a PhD in molecular and medical genetics at the University of Toronto in Canada, where she was trained by Mike Tyers. Increasingly, Liz’s research goals moved towards using developmental biology insights to understand cancer mechanisms, and she was introduced to zebrafish as a powerful tool for such translational studies by Leonard Zon. Between 2001 and 2004 Liz was a postdoctoral fellow in the Zon lab at Harvard Medical School, where she made important discoveries about the role of BRAF kinase in melanoma development. She was then awarded an MRC fellowship at the University of Oxford, and is now a Senior Lecturer and MRC Career Track Scientist at the MRC Institute for Genetics and Molecular Medicine at the University of Edinburgh. Liz is a member of the Young Academy of Scotland at the Royal Society of Edinburgh and, in addition to other editorial roles, has recently joined *Disease Models & Mechanisms* (DMM) as a Monitoring Editor.

**What inspired your interest in genetics? Have you always wanted to be a scientist?**

There was never a moment when I decided I wanted to become a scientist. I’ve liked science since school – but I also liked lots of subjects – and my grades were good. But back then I didn’t really have a clear idea of what a scientist does. I always liked asking questions, and I ended up doing a course in genetics as an undergraduate at Dalhousie University. Although all my science subjects were taught at Dalhousie, I was actually a joint student at King’s College, one of Canada’s oldest universities, which specializes in theology, philosophy and journalism. Students had access to a particularly beautiful library, and I clearly remember sitting there one day, studying for genetics, reading a textbook by David Suzuki that contained an amazing figure of a bacterial chromosome. I was fascinated by the prospect of engineering and genetically modifying a bacterial chromosome, and I realized then that genetics tells us amazing things.

**Figure:**
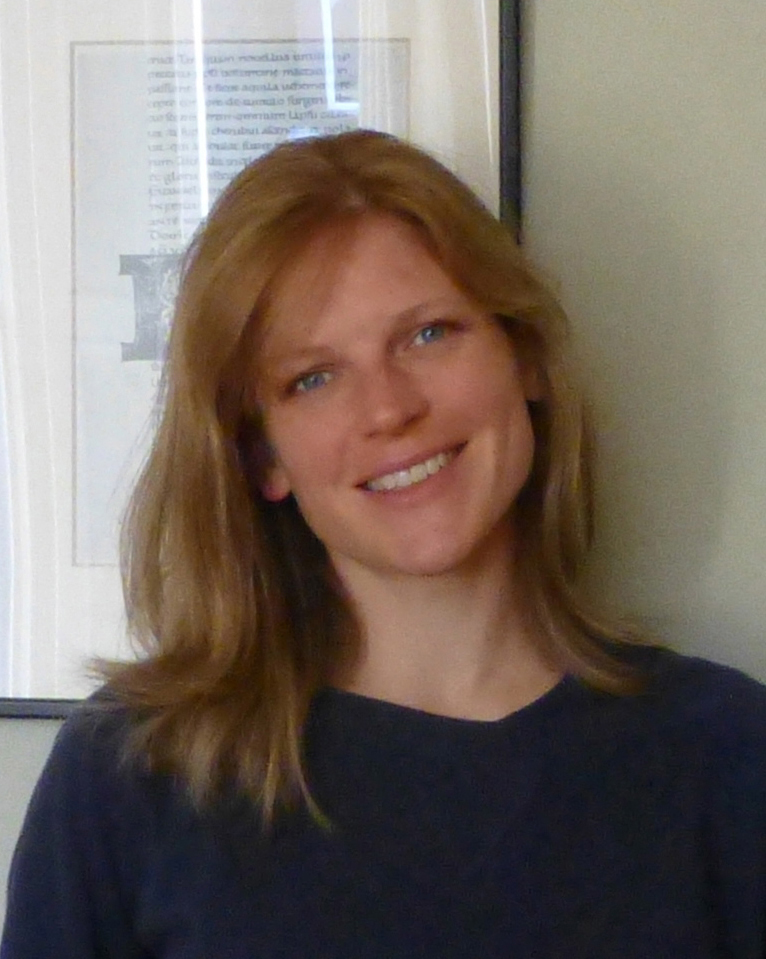


So, without really having a career path in mind, I did an undergraduate honors project in Microbiology with Gerald Johnston, who had trained with Lee Hartwell [Nobel prize winner for his work on cell cycle control]. When you work with somebody like that as an undergraduate, research is incredibly exciting. I very quickly realized that I loved being in the lab; I loved being with people who are also interested in asking questions and I loved the independence. I remember thinking that the graduate students were so lucky because they could stay in the lab to do experiments for as long as they wanted. Even though I had other commitments and classes, I found myself staying very late in the lab to cram it all in and do experiments as thoroughly as possible. As part of my project, I did a high-copy suppressor screen with yeast to identify regulators of exit from stationary phase and entry into the cell cycle.

**How did your transition from basic to applied science come about?**

I did my PhD at the Samuel Lunenfeld Research Institute in Toronto, with Mike Tyers [Michael Tyers, now based at University of Montreal]. I’d completed a rotation project looking at the cell cycle in budding yeast in Mike’s lab, and also a rotation project with Joseph Culotti, who uses the nematode worm to understand neuronal development. I was torn between the two, but I ultimately decided to go for the yeast cell cycle project – yeast were my first love! I always felt that we were using the model to understand cancer in the long run, which was a real driver for me. Even though we were doing basic science, I knew that the work had implications for understanding the pathways relevant to cancer biology.

It was a really productive time; yeast models were at the forefront for understanding the cell cycle machinery. Mike is very creative and ambitious, and it was very exciting to work on big questions in the field. We worked really hard in Mike’s lab – lots of late nights coupled with excellent science. Tony Pawson’s lab was right next door, and Janet Rossant and Alan Bernstein’s labs were on the floor below. All the labs around us were discovering fundamental aspects of biology that were clearly important in a disease context. Some of our key findings included the identification of E3 ubiquitin ligases that control the yeast cell cycle and metabolism. We figured out how G1 cyclins are regulated by degradation during the cell cycle and that multi-protein complexes (SCF complexes) help target the G1 cyclins and transcriptional regulators to the ubiquitin-mediated degradation pathways.

Around that time, Mike met Len Zon, who was doing screens in zebrafish to identify genetic mutants that affected the cell cycle. Mike felt that Len’s lab would provide a great opportunity for me as a postdoc. Len’s approach was to screen for embryonic cell cycle phenotypes, and then ask whether the implicated gene could contribute to cancer biology in the adult fish. Mutants provide a tractable system for asking a range of questions, and having a clear embryonic phenotypic readout facilitates small-molecule screening and suppressor isolation. I was really inspired by Len’s work, so I went to see him, and we both agreed that his lab would be a good fit for me. I started off screening for cell cycle mutants, and ended up generating the first BRAF model for melanoma in zebrafish. Melanoma is the most deadly form of skin cancer, and we showed that activation of a mutant BRAF, carrying a mutation that had been identified by sequencing in human melanoma, was one of the primary events in melanoma development.

**What are the main advantages of using the zebrafish as a model system for cancer?**

The ability to do genetic screens to look for new pathways that tell you about fundamental aspects of developmental and cell biology, and to apply the findings to tumors, is very powerful. The transgenic technology for zebrafish is robust and well established, allowing us to rapidly analyze some of the massive amounts of data that are coming out of human cancer studies. Work coming out of the Zon and Look [A. Thomas Look, Harvard University] labs really demonstrates the potential to use zebrafish for rapid and effective functional genomics studies in cancer.

The potential for intravital imaging is obviously a major advantage. Being able to visualize what’s happening in the tumor is really important, not least because it allows us to directly assess the impact of potential therapies on cancer cells and at the whole-organism level. We don’t need to cut holes as we do in mice – we can just ask questions and then observe. There aren’t many model systems that allow that. Also, there are other models, like the worm and the fly, that are just as experimentally tractable, but the zebrafish is unique in that its tumors actually look like human tumors. The tumor microenvironment is similarly complex to that in humans, so we can look at the impact of neighboring cells on cancer development. This makes zebrafish a great complementary model to mice and other organisms, but ultimately we need to rely on multiple models to get the bigger picture in cancer research.

“We don’t need to cut holes [in zebrafish] as we do in mice – we can just ask questions and then observe – there aren’t many model systems that allow that”

**What is your lab currently working on?**

We’re interested in understanding the cellular and developmental mechanisms that contribute to melanoma. We perform small-molecule screens, particularly focused on melanocyte stem cells, with a view to identifying new processes with a role in melanoma progression. Our general approach is to identify developmental phenotypes and then look to see whether the pathway is altered in the context of melanoma.

We’re also developing small-molecule screens with the aim of targeting melanoma cells for cell death. Recently, we’ve been working on target identification for a range of compounds, including nitrofurans, and we’re now trying to use our findings to target subpopulations of tumor cells. In these screens, we’re stepping away from zebrafish somewhat by relying on primary human melanoma lines. So, we exploit zebrafish for discovery and to understand how a compound works – for example by analyzing structure-function relationships – and then apply this information in human cells. Using this approach, we’ve made a small molecule that’s potent against melanocytes but doesn’t cause toxicity at the whole-animal level. We’re now working on the pre-clinical studies that are needed to launch this, and other promising compounds, into the clinical arena.

**What’s been your most exciting breakthrough to date?**

I would never limit it to just one, but there are a few moments that are particularly etched in my brain. One came as a graduate student in Mike’s lab: I’d been trying to find Cdc53 interaction proteins by two-hybrid screening and, one day, the sequencing data showed that it had finally happened. Back in those days, getting sequencing data back and being able to do BLAST searches to identify the genes was exciting in itself. Another time, we identified SCF E3 ubiquitin ligase complexes and we were able to show that F-box proteins dictated the substrate specificity *in vivo* – this was a real breakthrough. We showed that, in yeast, Cdc34 (the E2) and Cdc53 and Skp1 (parts of the E3) were components of the core complex for ubiquitylation of substrates, but the F-box protein was the key to substrate specificity. I remember processing a northern blot that confirmed this and running back from the dark room to Mike’s office to share the news. Then in Len’s lab, there was the moment that I first saw nevi – in the form of little black spots – on fish that had been injected with oncogenic BRAF. Len was in a meeting, and I needed to leave the lab to pick up my daughter, so I left the fish in the tank on my bench, and a note for Len saying ‘Go look at the fish!’.

Since setting up my own lab, there have been a lot of exciting moments. A notable one was being able to recapitulate human cardiofaciocutaneous [CFC] syndrome phenotypes in zebrafish expressing specific BRAF and MEK alleles, and showing that the phenotypes can be rescued using small-molecule inhibitors. This was an exciting and emotionally powerful discovery, because in this project we work closely with the families of affected individuals and the potential real-world impact of the findings are more palpable. Another was when we identified the target of a small molecule *in vivo* using zebrafish. The thrill of discovery is what keeps scientists going, especially when we’re on the road to being able to provide cures and better therapies. The intellectual stimulation and being around people with similar goals is also rewarding – building a strong network of collaborators is important. The science is difficult, and you need to keep communicating with and sharing your ideas with peers. I have a great team, and we are always discussing ideas and data.

“The thrill of discovery is what keeps scientists going, especially when we’re on the road to being able to provide cures and better therapies”

**In the zebrafish community, do you think there is enough dialog between the basic scientists and the clinicians?**

I think the zebrafish field is strong in this respect, in part because of the prominence of researchers who are clinical scientists. Many of the principal investigators are MD PhDs, and many of the basic scientists have a good understanding of disease, and there is a lot of discussion between the two groups. Cancer and immunity are two key areas for translational research using the zebrafish, but the value of the model is being increasingly recognized in other fields, such as pancreatic disease, neurological disease and behavioral disorders.

The genetic toolkit for zebrafish is very well developed and accessible to the medical world in a way that isn’t yet the case for other fish species. Model systems that are relatively easy and don’t need extensive development facilitate the ability for clinical questions to be addressed immediately. This is especially true when it comes to the challenge of sorting out a large number of disease-causing alleles. Also, with zebrafish there is a critical mass of scientists with the right expertise and tools to ensure that experimental standards are maintained across the community. As we sequence more and more human genomes and identify further disease-related polymorphisms, I think there will be even more dialog between the basic and clinical scientists who use zebrafish.

**What would you say are the three most urgent challenges in the cancer field?**

Metastases are an important clinical problem because, whereas primary tumors can often be surgically excised, secondary tumors are usually fatal. Another major issue to address is the recurrence of tumors after drug treatment. In melanoma, for example, treatment with BRAF inhibitors can have a dramatic effect, but relapse is discouragingly common. Understanding how relapse occurs and finding out which cell types should be targeted is important in tackling this problem.

I think that in medicine we need to think more about prevention of cancer, not only of treatment. We know so much about what can cause and contribute to cancer, yet healthcare systems are geared towards treating it rather than preventing it. We know that exercise, diet and certain lifestyle choices are important in cancer risk – how can we more effectively use this information to reduce the cancer burden through preventative measures? This is a bit of a contentious issue as there is obviously no guarantee that eating well, exercising, not smoking, etc. will eliminate an individual’s risk of developing cancer later in life, but maybe a greater degree of individual responsibility and awareness is needed. Also, population-level studies with animal models could be useful for assessing the effect of the environment on disease risk. Most of the current animal models involve driver mutations with strong effects, but the impact of polymorphisms and gene-environment interactions isn’t always easy to model – this is a real challenge.

Another challenge is to generate affordable medicines on a long-term basis. I’m inspired by David Lane [British oncologist best known for his discovery of p53 tumor suppressor], who emphasizes the importance of academic drug discovery in the development of affordable drugs. There is a growing body of knowledge in the academic community about how compounds work and what their targets are – it’s important to not get discouraged by the perceptions of the pharmaceutical world about what is and isn’t interesting and worth pursuing. In an academic setting, we have opportunities to identify new targets for known drugs – a process known as drug repurposing – which provides a powerful, economical way to generate new treatment strategies. Model systems provide useful tools to assess the effects of established compounds in different clinical situations.

“…it’s important to not get discouraged by the perceptions of the pharmaceutical world about what is and isn’t interesting and worth pursuing”

**How did you become involved with DMM?**

In 2010 I published one of my favorite studies in DMM. In this study, we collaborated with Mike Tyers and combined zebrafish phenotypic chemical screens with yeast chemical genetics profiling to explore gene-environment interactions that modulate melanocyte pigmentation. We were looking for a journal that would recognize the value of the study as a unique model system approach providing insight into disease mechanisms, and DMM provided the ideal forum.

My group also has an ongoing collaboration with a clinical group led by Kate Rauen (UCSF) working on CFC syndrome, and we published some of the work from this project in DMM. We’d shown previously that treatment with potent MEK inhibitors can override the deleterious effects of CFC mutations in zebrafish. However, these inhibitors are designed as anti-cancer treatments and they can have dramatic developmental effects, so we wanted to find out whether low doses of the compounds could be effective in treating CFC syndrome. We were thrilled to find that partial inhibition of the target pathway with a continuous low-level dose of MEK inhibitors can control the disease gene effects in zebrafish, with minimal negative side effects. When establishing new therapies, there needs to be a trade-off between efficacy and toxicity, so it’s important to look at the effects of drugs at the whole-animal level – this kind of holistic approach is appreciated in DMM. The journal’s aims align with my own, as I really believe that model systems can help us to develop new and improved drugs, so when Ross [Cagan, Editor-in-Chief] approached me to take on the role of monitoring editor, I was happy to accept.

**What advice do you give to students thinking of pursuing a career in research?**

I don’t often offer much advice on their career path, because I think that this is shaped by very individual events and factors. But I do emphasize the things that I love about science; for example, the international nature of the career. We are lucky to be able to work with individuals with similar goals and outlooks from all over the world. I encourage graduate students to embrace the opportunities to travel to and live in different places and to meet new people. Compared with many careers, these are rare and special opportunities, and I think it’s vital to jump on them early on in life, before life can become more complicated family-wise. I also value the feeling of being part of a team to which I can make a real contribution – even as an undergraduate doing a lab project, I felt that my ideas and opinions really counted.

**How do you achieve a healthy work-family balance?**

My husband is also an academic as a specialist in ancient Greek literature and papyrology, and my children inherently work hard to achieve their goals, so as a family we have a shared vision and are supportive of each other. My daughter is 13 and is passionate about literature and theatre, and, my son, who’s 9, is very competitive in tennis: I’ve learned a lot from them in terms of the way they cope with pressure and setbacks, as well as success. Having children never feels like a burden, and we make the most of our time together outside work. One of our favourite things to do together on Friday nights is to watch movies and TV programs, particularly comedy shows – laughing together is important.

There are periods when the work-family balance is skewed, but the balance always comes back. Being able to communicate by email, phone and text is obviously helpful – my students can contact me even when I’m not in the lab. There are ways of bringing the two together too; for example, I have helped establish zebrafish tanks as an educational tool at my son’s school. We used different types of fish to study inheritance, which proved to be a lot of fun for both the children and me.

